# Some Thoughts and Experiments upon Erosion

**Published:** 1886-05

**Authors:** Edgar D. Swain

**Affiliations:** Chicago, Ill.


### Independent Practitioner.

Vol. VII. May, 1886. No. 5.

### Original Communications.

Note.—No paper published or to be published in another journal will be accepted for this department. All papers must be in the hands of the Editor before the first day of the month preceding that in which they are expected to appear. Extra copies will be furnished to each contributor of an accepted original article, and reprints, in pamphlet form, may be had at the cost of the paper, press-work and binding, if ordered when the manuscript is forwarded. The Editor and Publishers are not responsible for the opinions expressed by contributors. The journal is issued promptly, on the first day of each month.

### SOME THOUGHTS AND EXPERIMENTS UPON EROSION.

BY EDGAR D. SWAIN, D. D. S., CHICAGO, ILL.

(Concluded from page 184.)

In my last paper upon the subject of Erosion I presented a theory borrowed from experiments made by Mr. Kincely Bridgman, for the purpose of proving that dental caries was the result of an electro-chemical process, and that the results of those experiments, repeated by myself, gave hopes of a solution of the causes producing that disease. I then promised to experiment further in this direction, and to report progress at some future time. Immediately after, I had prepared by a chemist solutions of the following acids, barely strong enough to give an acid reaction with litmus. Aiming to secure, as near as possible, the acidity detected in the oral secretions, when present at all, I procured ten small glass vessels, and cemented two teeth to the bottom of each, crowns up; also with them a piece of copper wire. These were then filled with the acid solutions, covering the teeth, so that about one-half the enamel was submerged. Each glass was then covered with a sheet of paraffine paper, tightly bound to it, to prevent evaporation and exclude the dust. These vessels were placed upon a shelf built against a chimney, warmed by a continual fire. A thermometer hanging upon the chimney varied at no time more than a few degrees from the temperature of the oral cavity (98° F.). From time to time, as necessary, the acids were tested with litmus, and replenished to preserve a medium strength and quantity. These experiments were continued from early in October to the following May, when a careful examination of the contents of each glass was made, verifying the strength of the acid solutions used, and the effect upon the teeth and wire noted.

The results were as follows:

Oxalic Acid—Strength, $\frac{1}{260}$ of one per cent. No action upon teeth or wire.

Citric Acid—Strength, $\frac{1}{130}$ of one per cent. No action.

Phosphoric Acid—Strength, $\frac{1}{130}$ of one per cent. Slight roughening of all that part of the enamel immersed. No apparent action upon the cementum. Wire slightly constricted at the line of immersion.

Lactic Acid—$\frac{1}{60}$ of one per cent. Marked groove, and softened enamel at the line of immersion.

Acetic Acid—Strength, $\frac{1}{60}$ of one per cent. No action.

Butyric Acid—Strength, $\frac{1}{30}$ of one per cent. No action.

Tannic Acid—Strength, $\frac{1}{30}$ of one per cent. No action, but a slight brownish color of cementum.

Tartaric Acid—Strength, $\frac{1}{260}$ of one per cent. No action.

Sulphuric Acid—Strength, $\frac{1}{130}$ of one per cent. Enamel roughened over its entire surface; more so beneath the liquid. Light deposit of sulphate of lime above the immersed part. Cementum softened; wire constricted at the line of immersion.

Nitric Acid—Strength, $\frac{1}{130}$ of one per cent. Enamel roughened over its entire surface. Cementum not visibly affected. Wire eroded over its entire surface, but more so just above the surface of the liquid, where in contact with the atmosphere.

Hydrochloric Acid—Strength, $\frac{1}{140}$ of one per cent. Entire enamel roughened. No apparent action upon the cementum. Wire constricted at the point of contact of liquid and atmosphere.

And just here, though foreign to the subject, I call attention to the difference in diffusibility of the acids used, those which we consider the weakest of the acids requiring a very much larger quantity of water to reduce them to that condition represented by their action upon litmus.

The reader will remember that the results of the experiments reported in my previous essay were very marked, and were only made, as were Mr. Bridgman's, with sulphuric acid, and that of a strength far greater than those used and reported above. In fact, the strength was sufficient to assure us, not only action upon the metal used, but also upon the lime salts of the teeth.

These last experiments have had a tendency to weaken my faith in the theory that electro-chemical action is responsible for the conditions we observe upon the teeth, known as erosion. Therefore, for some years I have been continuously upon the look-out for some more plausible theory to account for this disease. I must admit, however, that I am still as deep in ignorance as ever.

As a matter of course, I have observed carefully all cases of erosion presenting themselves in my own practice, and have been greatly assisted in these observations by my brother practitioners, and I now take occasion to thank Dr. Geo. H. Cushing, of Chicago, and Dr. G. V. Black, of Jacksonville, Ills., for valuable assistance.

The close and long continued study of this affection of the teeth has brought to notice such numerous and varied conditions and effects that I am almost in despair, and the hope that what I may say will call forth the views of other observers is my only excuse for allowing these papers to appear in print. I now feel satisfied that erosion is not a result of electro-chemical action, neither is it to be attributed to mechanical forces. The use of the tooth brush cannot be a cause where it has never been employed. The uses of acid fruit, or acids with our food, sudden changes of temperature, and all the other theories of this nature must, to my mind, be abandoned, and new theories sought for.

At one time I was strongly inclined to believe that Prof. Black, in his lectures upon the causes of caries, had given me a leader in his theory of a digestive secretion, but more careful considerations of all the conditions presented have only doomed me to further disappointment.

I have carefully studied the development of the teeth, in hopes to find here a path which would lead me out of this tangled depth.

Were it a fact that the teeth were developed simultaneously, we could attribute the lines of erosion on the same plane to a defect in calcification, or a result of any disturbance causing a temporary arrest of development. A reference to the "Calcification Chart," prepared by Prof. Black, shows at once that a stable theory cannot thus be established.

Magitot believed infantile convulsions (eclampsia) to be a common cause of erosions. It seems to me, however, he must be mistaken, for the same reasons which apply to imperfect development.

Hutchinson believes syphilis to be responsible for this disease, but here again we meet the same objections, reinforced by the knowledge that syphilis is a continuous disease, and if it be inherited and the teeth afflicted thereby, it would involve the entire organ, from formation of the enamel cap to the completely developed tooth.

In fact, circumstances do not warrant us in assuming that any disease causing an arrest of development would leave the tissues in such a condition that, if a central incisor be affected across the middle of its crown horizontally, five or six years later other teeth, say the cuspids or bicuspids, would be similarly affected.

In a recent conversation with Prof. Black, he said:—"I do not consider Erosion a species of caries, as some have suggested, but do believe it to be the result of abnormal secretions, from glands situated in the lips and tongue, at the points of contact with the teeth." The lips touch most forcibly the highest point of the convexed surface of the teeth, leaving above that point of contact a pocket. The glands of the lip at point of contact may become closed, and consequently a low condition of inflammation exist, causing abnormal secretions. This, however, will not account for the disease upon the lingual surface of the superior incisors, and I have never yet seen a case of Erosion upon the lingual surface of the inferior incisors.

Erosion is probably a result of combined conditions, and we are not justified in attributing all cases to any one apparent cause. The study of this disease opens up a large field for observation, which should be developed by the young men of our profession, and followed to a successful termination.

I have consumed much space in telling what I do not know, but hope others will continue the investigations until, like "Pyorrhœa Alveolaris," we shall be able to successfully arrest its ravages.

